# Pituitary Hyperplasia and Oncocytic Thyroid Neoplasia in a Child With Severe Hypothyroidism

**DOI:** 10.1016/j.aed.2025.11.004

**Published:** 2025-11-13

**Authors:** Liany F. Acosta-Paguada, Milca S. Velásquez-Hernandez, Paola Sophia Bonilla Medina, Carol Josseline Zuniga Garcia, Eduardo Smelin Perdomo Domínguez

**Affiliations:** 1 Faculty of Medicine, Catholic University of Honduras, San Pedro Sula, Honduras; 2 Pediatric Endocrinologist, Hospital Bendaña, San Pedro Sula, Honduras; 3 Pediatric Endocrinologist, Hospital Mario Catarino Rivas, San Pedro Sula, Honduras; 4 Pediatric Neurologist, Hospital Mario Catarino Rivas, San Pedro Sula, Honduras; 5 GIMUNICAH, Faculty of Medicine, Catholic University of Honduras, San Pedro Sula, Honduras

**Keywords:** hypothyroidism, macroadenoma, oncocytic thyroid carcinoma, pituitary hyperplasia

## Abstract

**Background/Objective:**

Severe pediatric hypothyroidism may cause growth failure and pituitary hyperplasia mimicking adenoma. Although thyroid nodules are rare in children, their malignancy risk is higher than in adults. We report a boy with severe hypothyroidism, growth failure, pituitary hyperplasia, and oncocytic thyroid carcinoma, underscoring the need for comprehensive evaluation.

**Case Presentation:**

A 10-year-old boy presented with growth delay, height 105 cm (<1st percentile, −6.7 SD), and weight 21.2 kg (third percentile, −2.03 SD). Examination revealed pallor, dry skin, alopecia, Tanner stage I/I, and bitemporal hemianopsia. Laboratory results showed thyroid stimulating hormone >500 mIU/ml (reference 0.45–4.50 mIU/L), free thyroxine <0.30 ng/dl (reference 0.93–1.60 ng/dl), prolactin 102 ng/ml (reference 5–20 ng/ml), and insulin-like growth factor 1 of 8 ng/ml (reference 123–497 ng/ml). Pituitary magnetic resonance imaging demonstrated a homogeneous, isointense sellar lesion with optic chiasma displacement. Thyroid ultrasound showed a 43.9 mm hypoechoic nodule, and fine-needle aspiration classified it as Bethesda category IV. Total thyroidectomy confirmed encapsulated angioinvasive oncocytic carcinoma, followed by radioactive iodine therapy. With levothyroxine and growth hormone treatment, pituitary hyperplasia regressed, and height increased by 15 cm in 1 year.

**Discussion:**

Pituitary hyperplasia secondary to hypothyroidism is reversible with levothyroxine and must be differentiated from adenomas to avoid unnecessary surgery. Oncocytic thyroid carcinoma is rare in children, and its coexistence with hypothyroidism is unusual.

**Conclusion:**

This case emphasizes the importance of thyroid function testing in children with growth failure and sellar lesions, and vigilance in evaluating pediatric thyroid nodules given their elevated risk of malignancy.

**Highlights**

• Thyroid function evaluation is key in children with growth delay and pituitary lesions
• Pituitary hyperplasia may mimic adenoma but regresses with levothyroxine therapy
• Pediatric thyroid nodules carry higher malignancy risk than in adults
• Severe hypothyroidism alters multiple hormonal and metabolic pathways

**Clinical Relevance**

A 10-year-old boy with severe hypothyroidism presented with growth failure, pituitary hyperplasia mimicking macroadenoma, and oncocytic thyroid carcinoma. This case highlights the importance of thyroid testing in children with growth delay and the need for thorough evaluation of pediatric thyroid nodules due to their higher malignancy risk.

## Introduction

Severe hypothyroidism in children may present with growth failure and, when prolonged, can lead to pituitary hyperplasia that mimics adenomas on imaging.^1^^,^^2^ Recognition of this entity is crucial, as it typically regresses with thyroid hormone replacement, avoiding unnecessary neurosurgical interventions.^3^

Thyroid nodules are rare in the pediatric population but carry a substantially higher risk of malignancy than in adults.^4^ Among these, oncocytic thyroid carcinoma (OTC) is an uncommon histologic subtype, with only sporadic pediatric cases reported.^5^

Here, we present the case of a Honduran boy with severe, long-standing hypothyroidism manifesting as growth failure, pituitary hyperplasia mimicking macroadenoma, and the concomitant diagnosis of pediatric OTC—a rare coexistence that emphasizes the importance of comprehensive evaluation in such patients.

## Case Report

We report the case of a 10-year-old Honduran boy born to nonconsanguineous parents. He is the second of 3 children born to a 28-year-old mother. The pregnancy was uneventful, with no known exposure to teratogenic drugs, infections, or radiation. He was delivered vaginally without complications, with a birth weight of 5.4 kg (large for gestational age). His past medical history was notable only for bronchial asthma treated with beclomethasone and salbutamol; he had no history of surgeries, hospitalizations, or allergies. Family history revealed breast and lung cancer in the maternal grandmother, while both parents were healthy.

According to his mother, growth delay was first noticed at age 4, leading to medical evaluation and regular follow-up that lasted until the COVID-19 pandemic, when medical care was interrupted. At his initial presentation at age 10 years, his vital signs were within normal limits. His weight was 21.2 kg (third percentile, −2.03 SD), height 105 cm (<1st percentile, −6.7 SD), and body mass index 19.23 kg/m^2^ (50th percentile, −0.02 SD), with a projected adult height of 134.6 cm. On examination, he appeared pale, with dry skin, localized alopecia, Tanner stage I/I, and a shawl scrotum. Ophthalmologic evaluation revealed bitemporal hemianopsia, consistent with optic chiasma compression. Neck examination revealed a diffusely and asymmetrically enlarged thyroid gland, with predominance of the left lobe, which was occupied by a firm, well-circumscribed, non-tender mass approximately 4-5 cm in diameter. No cervical lymphadenopathy was palpated, and no thyroid bruit was auscultated.

Initial laboratory tests showed normal hematology, but revealed several significant abnormalities, including severe hypothyroidism (thyroid-stimulating hormone (TSH) >500 mIU/L; reference 0.45–4.50 mI U/L; free T4 <0.30 ng/dl; reference 0.93–1.60 ng/dl), hyperprolactinemia (102 ng/ml; reference 5–20 ng/ml), markedly low insulin-like growth factor-1 (IGF-1) (8 ng/ml; reference 123–497 ng/ml), low IGF insulin-like growth factor-binding protein-3 (IGFBP-3) (0.949 mg/L; reference 1.4-5.2 mg/L) vitamin D insufficiency (15.5 ng/ml; reference 12-20 ng/ml), total cholesterol 285 mg/dl (reference total cholesterol < 200 mg/dl), low-density lipoprotein 173 mg/dl (reference <130 mg/dl), triglycerides 134 mg/dl (reference <100 mg/dl), and elevated transaminases (aspartate aminotransferase 85 U/L, ALT 70 U/L; reference aspartate aminotransferase 18-36 U/L, ALT 9-25 U/L). Antithyroid antibodies (anti-TPO and antithyroglobulin) were negative. Bone age, assessed by Greulich and Pyle standards, was consistent with 1 year 6 months, confirming severe delay relative to chronological age.

**Fig. 1:**
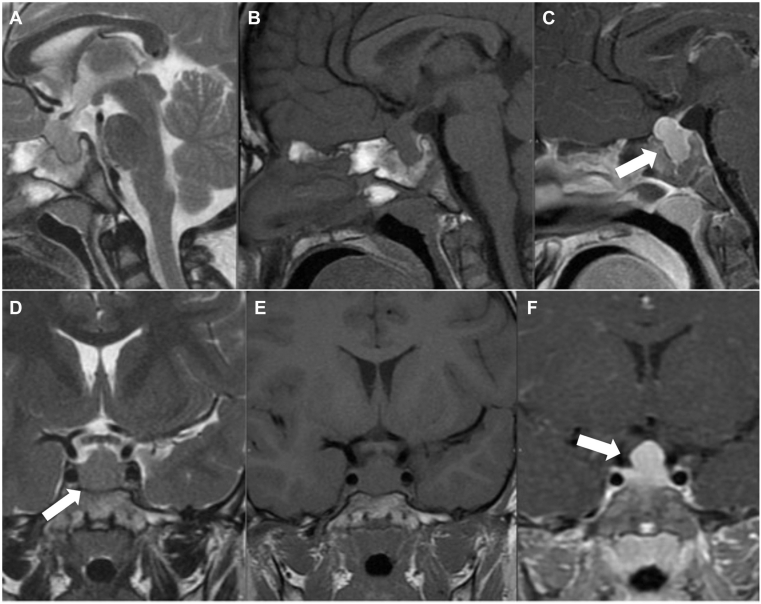
Initial pituitary MRI. (A–C) Sagittal T2, T1, and T1 postcontrast sequences show diffuse homogeneous enhancement of an isointense sellar lesion (white arrows) with mild upward displacement of the optic chiasm. (D–F) Coronal T2, T1, and T1 postcontrast dynamic sequences demonstrate a centrally located pituitary stalk with contact of the optic chiasm (white arrows). MRI = magnetic resonance imaging.

Pituitary magnetic resonance imaging (MRI) revealed a homogeneous, isointense lesion measuring 17 × 9.8 × 10.5 mm occupying the sella turcica, with mild upward displacement of the optic chiasm (Fig. 1). Initially interpreted as a macroadenoma, it was subsequently reconsidered as pituitary hyperplasia secondary to severe primary hypothyroidism. Thyroid ultrasound demonstrated a heterogeneous, hypoechoic, circumscribed nodule with increased vascular flow in the left lobe, volume 11.8 cc, classified as ACR TIRADS IV. Fine-needle aspiration confirmed an oncocytic follicular neoplasm, Bethesda category IV. Chest computed tomography excluded metastatic disease. Differential diagnoses included pituitary adenoma versus hyperplasia and benign versus malignant follicular neoplasm. The overall clinical, radiological, and pathologic correlation favored pituitary hyperplasia secondary to severe hypothyroidism associated with oncocytic thyroid carcinoma.

**Fig. 2:**
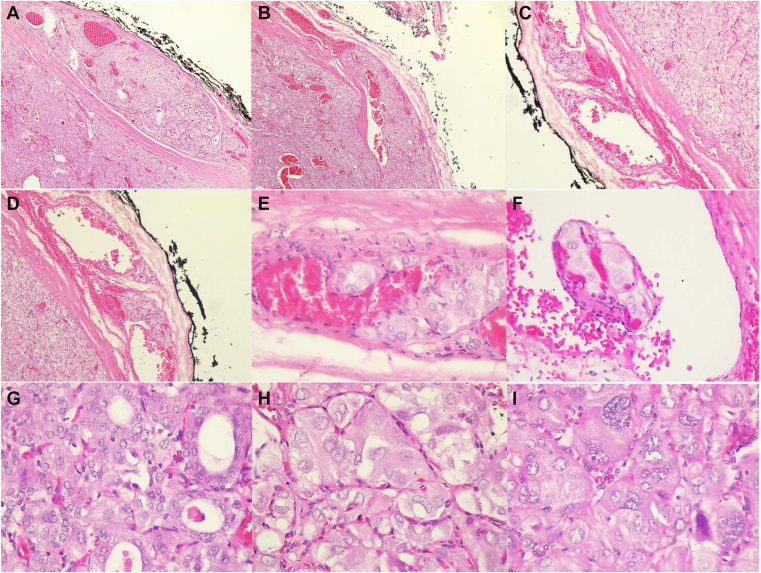
Oncocytic (Hürthle cell) carcinoma of the thyroid. (A–D). Encapsulated lesion showing multifocal capsular and vascular invasion. (E–F) Intraluminal oncocytic nests within capsular vessels, partially lined by endothelium. (G–I) Oncocytic cells with granular eosinophilic cytoplasm and enlarged irregular nuclei with vesicular chromatin, prominent nucleoli, and occasional pseudoinclusions.

Treatment was initiated with levothyroxine (LT4) 25 mcg/d and vitamin D supplementation (1000 IU/day), with progressive adjustment to 75 mcg/day based on clinical and laboratory response. Following confirmation of an oncocytic follicular neoplasm, the patient underwent total thyroidectomy with lymph node dissection, without intra- or postoperative complications. Histopathology revealed an encapsulated angioinvasive oncocytic carcinoma of the left thyroid lobe (3.5 × 2.2 × 2 cm), with lymphovascular invasion, negative surgical margins, and no extrathyroidal extension; the right lobe was hypoplastic (Fig. 2). Adjuvant therapy with radioactive iodine (100 mCi I-131 orally) was subsequently administered, with good tolerance and a negative whole-body scan showing no evidence of residual or metastatic disease. Endocrine therapy was continued with LT4, alternating doses of 100 mcg and 75 mcg to achieve an effective average replacement dose. Due to persistent growth delay, growth hormone (GH) therapy was introduced at 0.7 mg nightly subcutaneously, later adjusted to 1.1 mg and then stabilized at 0.8 mg, together with LT4 100 mcg daily.

**Fig. 3:**
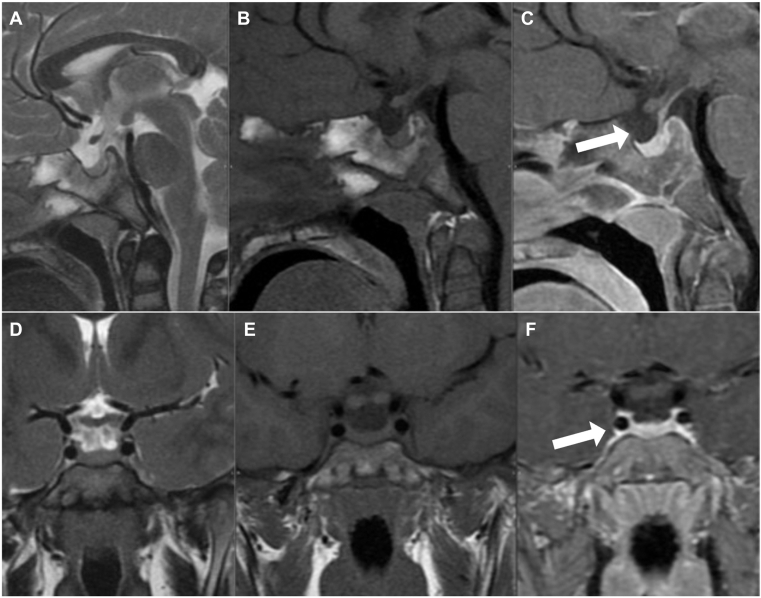
Follow-up pituitary MRI. (A–C) Sagittal T2, T1, and T1 postcontrast sequences show normal pituitary gland morphology (white arrows). (D–F) Coronal T2, T1, and T1 postcontrast dynamic sequences demonstrate a centrally located pituitary stalk without optic chiasm involvement (white arrows). MRI = magnetic resonance imaging.

During follow-up, initial thyroid function remained markedly abnormal (TSH 216.5 mIU/ml, free T4 0.42 ng/dl), but normalized under LT4 (TSH 2.52, free T4 1.32 ng/dl) with resolution of hyperprolactinemia (13.5 ng/ml). After surgery and radioiodine therapy, serum calcium remained stable (10.1 mg/dl; reference 9.2–10.5 mg/dl), with no hypoparathyroidism, and thyroid function tests showed fluctuations requiring therapeutic adjustments, with TSH ranging from 10.7–34.7 mIU/ml and free T4 between 0.1–1.75 ng/dl. Follow-up pituitary MRI revealed complete regression of the hyperplasia, with a normally defined pituitary gland and no displacement of adjacent structures (Fig. 3). Neck ultrasound confirmed total thyroidectomy without residual thyroid tissue.

**Fig. 4:**
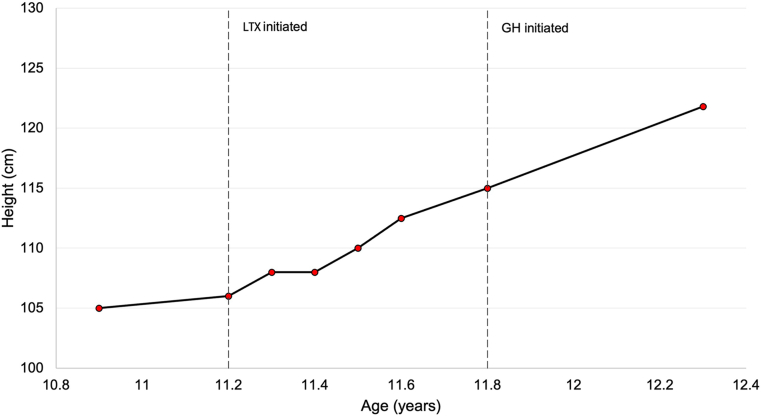
Growth trajectory over time. Height improved following initiation of levothyroxine (LTX) and subsequently growth hormone (GH), with an increase of approximately 15 cm within 1 year.

Clinically, the patient demonstrated significant improvement in linear growth, with a height increase of approximately 15 cm within 1 year after initiation of LT4 and GH therapy (Fig. 4). At his most recent evaluation, he remained clinically stable, under regular endocrinological follow-up, with no treatment-related adverse effects or evidence of disease recurrence.

## Discussion

This case exemplifies the systemic consequences of long-standing untreated hypothyroidism in childhood, manifested not only by pituitary hyperplasia mimicking macroadenoma but also by the unusual coexistence of OTC. Such an association is exceedingly rare and underscores the importance of comprehensive evaluation in children with growth failure.

Pituitary hyperplasia secondary to primary hypothyroidism has been widely reported in pediatric populations and may closely resemble macroadenomas on MRI, posing a significant diagnostic challenge.^2^^,^^3^ Chronic thyroid hormone deficiency stimulates thyrotroph proliferation, with histological studies estimating hyperplasia in 25% to 81% of patients, and the incidence rising to 70% when TSH levels exceed 50 μIU/mL.^4^^,^^5^ Regression after thyroxine replacement is well documented: approximately 85% of cases show pituitary shrinkage, usually evident within 2 to 4 months of therapy.^6^^,^^7^ Clinically, hypothyroidism in children typically causes poor linear growth and/or growth failure, and if left untreated may compromise adult height. Affected patients often present with pubertal delay, although precocious puberty has also been reported in severe long-standing cases.^8^ In our patient, complete involution of the pituitary lesion after LT4 therapy confirmed the diagnosis, thereby avoiding unnecessary neurosurgical intervention.

Hyperprolactinemia is another well-recognized finding in severe hypothyroidism. Although its pathogenesis is not fully understood, several mechanisms have been proposed, including excessive TRH stimulation of lactotrophs, impaired dopamine synthesis, reduced prolactin clearance, and altered gene expression in prolactin-secreting cells.^1^ Pituitary enlargement may further compress the stalk, impairing dopaminergic inhibition and contributing to prolactin elevation.^9^ Our patient also exhibited deficiencies in GH, IGF-1, and IGFBP-3, consistent with thyroid hormone–dependent regulation of the GH/IGF-1 axis. Hypothyroidism reduces circulating IGF-1 and IGFBP-3 levels and impairs GH secretion, abnormalities that typically improve after thyroxine replacement.^10^ These alterations likely accounted for the severe growth failure observed in this case.

Thyroid nodules are uncommon in children, with a prevalence of approximately 2%, but carry a malignancy risk of 18% to 26%, much higher than the 5% risk in adults.^11^ Pediatric thyroid cancers are also more frequently associated with lymph node involvement (40% to 80%) and distant metastases (up to 25%), although survival remains excellent, with 10-year overall survival exceeding 98%.^11^^,^^12^ In our case, biopsy of a left-sided thyroid nodule revealed OTC, a rare follicular-derived malignancy previously known as Hürthle cell carcinoma. OTC accounts for about 5.8% of pediatric thyroid cancers and has been reported in children as young as 7 years.^13^ Unlike the typical presentation as an asymptomatic thyroid nodule, our case occurred in the context of severe hypothyroidism, although a causal relationship between these 2 conditions cannot be established.

Molecular and genetic testing was not performed, which could have provided further insight into the possible relationship between hypothyroidism and oncocytic carcinoma. Moreover, causality between these 2 conditions cannot be established from a single case, and their coexistence may represent coincidental findings.

In conclusion, this case highlights the systemic impact of untreated pediatric hypothyroidism, including growth failure, pituitary hyperplasia, and metabolic derangements, while also documenting a rare association with OTC. Favorable outcomes were achieved through LT4 replacement, total thyroidectomy, adjuvant radioactive iodine, and endocrine optimization. Clinicians should maintain a high index of suspicion for thyroid disease in children with growth impairment and sellar lesions, and consider evaluation for thyroid nodules when clinical or imaging findings raise suspicion, as the risk of malignancy is substantially higher than in adults. Multidisciplinary care and vigilant follow-up are essential to optimize prognosis.

## Statement of Patient Consent

Informed consent was obtained from the patient's legal representative, and all procedures were performed according to the Declaration of Helsinki.

## Disclosure

The authors have no conflicts of interest to disclose.
